# Supplementary material for: Triacylglycerol synthesis enhances macrophage inflammatory function
Source: Nat Commun. 2020 Aug 14;11:4107. doi: 10.1038/s41467-020-17881-3 (PMC7427976; doi:10.1038/s41467-020-17881-3)
Supplement: Supplementary file 1 — Supplementary Information [file 41467_2020_17881_MOESM1_ESM.pdf]

Triacylglycerol synthesis enhances macrophage inflammatory function

Castoldi et al.,

Supplementary information

Supplementary Figure 1

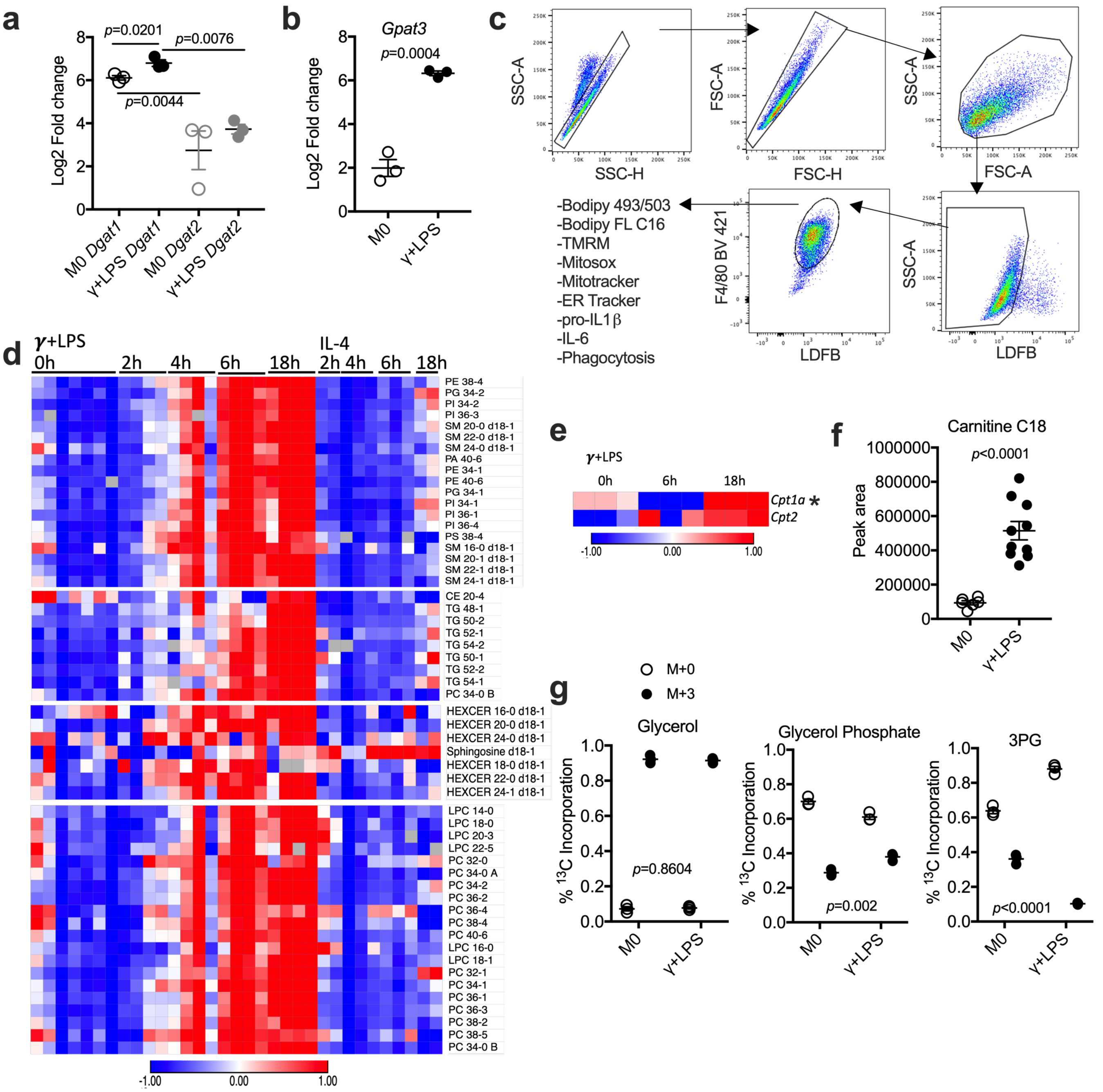

Supplementary Figure 1: Increased *Dgat1* gene expression, lipid content and incorporation of glycerol into TG synthesis in inflammatory macrophages.

(a) *Dgat1* and *Dgat2* gene expression in macrophages treated without (M0) or with IFNγ + LPS for 18 h. Data from RNAseq (n = 3 biologically independent samples). **(b)** *Gpat3* gene expression in macrophages treated without (M0) or with IFNγ + LPS for 18 h. Data from RNAseq (n = 3 biologically independent samples). **(c)** Representative gating strategy used to identify live, F4/80$^+$ macrophages and further staining as indicated. LDFB: Live/Dead fixable blue staining. **(d)** Time-dependent accumulation of different lipid species in macrophages after 0, 2, 4, 6 and 18 h of IFNγ + LPS or IL-4 stimulation. **(e)** *Cpt1a* and *Cpt2* expression in resting macrophages (0 h) and after 6 h or 18 h of stimulation with IFNγ + LPS (γ + LPS). Data from RNA-seq. **(f)** C18 acylcarnitine measured using MS in M0 macrophages and macrophages stimulated with IFNγ + LPS, for 18 h

(M0 n = 6, γ + LPS n = 10 biologically independent samples). **(g)** Fractional contribution of ^{13}C Glycerol (M+3) during a 30 min pulse of labeling resting macrophages (M0) or macrophages stimulated with IFNγ + LPS for 18 h (n = 3 biologically independent samples). Data are represented as mean values ± s.e.m. One-way ANOVA with Bonferroni's multiple comparison test **(a, g)**; Unpaired two-tailed Student's t test **(b, f)**. (*p < 0.05). (a-b) representative of one experiment. (d-g) representative of two experiments (c) representative of three experiments. Source data are provided as a Source Data file.

Supplementary Figure 2

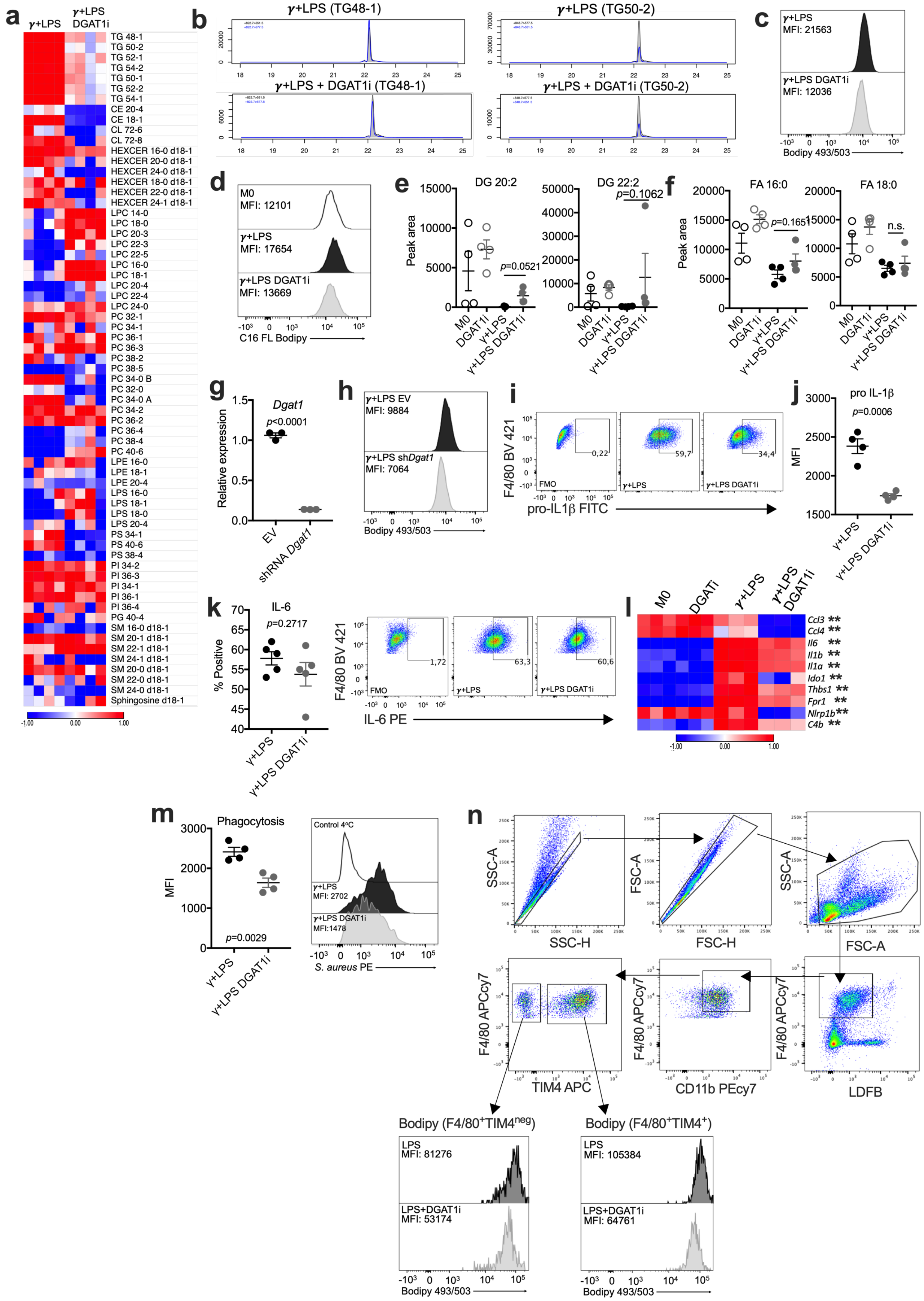

Supplementary Figure 2: Changes in lipid content of inflammatory macrophages as a result of loss of DGAT1 function.

(a) Lipidome at 18 h after addition of IFNγ + LPS without or with DGAT1i. **(b)** Representative mass spectrum (MS) of TG48-1 and TG50-2, x-axis: mass/charge ratio, y-axis: signal intensity. **(c)** Representative histogram of Bodipy 493/503 staining after 18 h of culture with IFNγ + LPS with or without DGAT1i. **(d)** Representative histogram of Bodipy FL C16 uptake after 18 h. **(e)** Effect of DGAT1i on DG (DG 20:2 and 22:2), and **(f)** FA (FA 16:0 and 18:0), in macrophages over 18 h of culture (n = 4 biologically independent samples). **(g)** Validation of ability of sh*Dgat1* to suppress *Dgat1* gene expression after 3 days of puromycin selection (n = 3 biologically independent samples). **(h)** Representative histogram of Bodipy 493/503 staining in macrophages transduced with empty vector or sh*Dgat1* and cultured with or without IFNγ + LPS for 18 h. **(i)** Representative pro-IL1β intracellular staining (values are percentage), and **(j)** pro IL-1β production, (MFI, median fluorescence intensity), 6 h after stimulation with IFNγ + LPS with or without DGAT1i (n = 4 biologically independent samples). **(k)** IL-6 production, percentage cytokine-positive F4/80$^+$ cells, 6 h after stimulation and representative IL-6 staining (n = 5 biologically independent samples). **(l)** Down regulated genes related to cytokine production and immune system processes after 18 h. **(m)** Effects of DGAT1i on the ability of inflammatory macrophages to phagocytose *S. aureus* (PE-labelled). Data show MFI for *S.aureus* fluorescence in F4/80$^+$ cells. Macrophages were stimulated with IFNγ + LPS for 18 h with or without DAGT1i and then incubated with PE-labeled *S. aureus* (PE) for 30 min (n = 4 biologically independent samples) and representative histogram. **(n)** Representative gating strategy to identify Bodipy 493/503 staining as indicated**, (**LDFB, Live/Dead fixable blue staining). Data are represented as mean values ± s.e.m. One-way ANOVA with Bonferroni's multiple comparison test **(e-f)**; Unpaired two-tailed Student's t test **(g, j, k, m)**. (***p* < 0.01). (a-b, e-f, n) representative of two experiments, (c-d, g-k, m), representative of tree experiments and (l) representative of one experiment. Source data are provided as a Source Data file.

Supplementary Figure 3

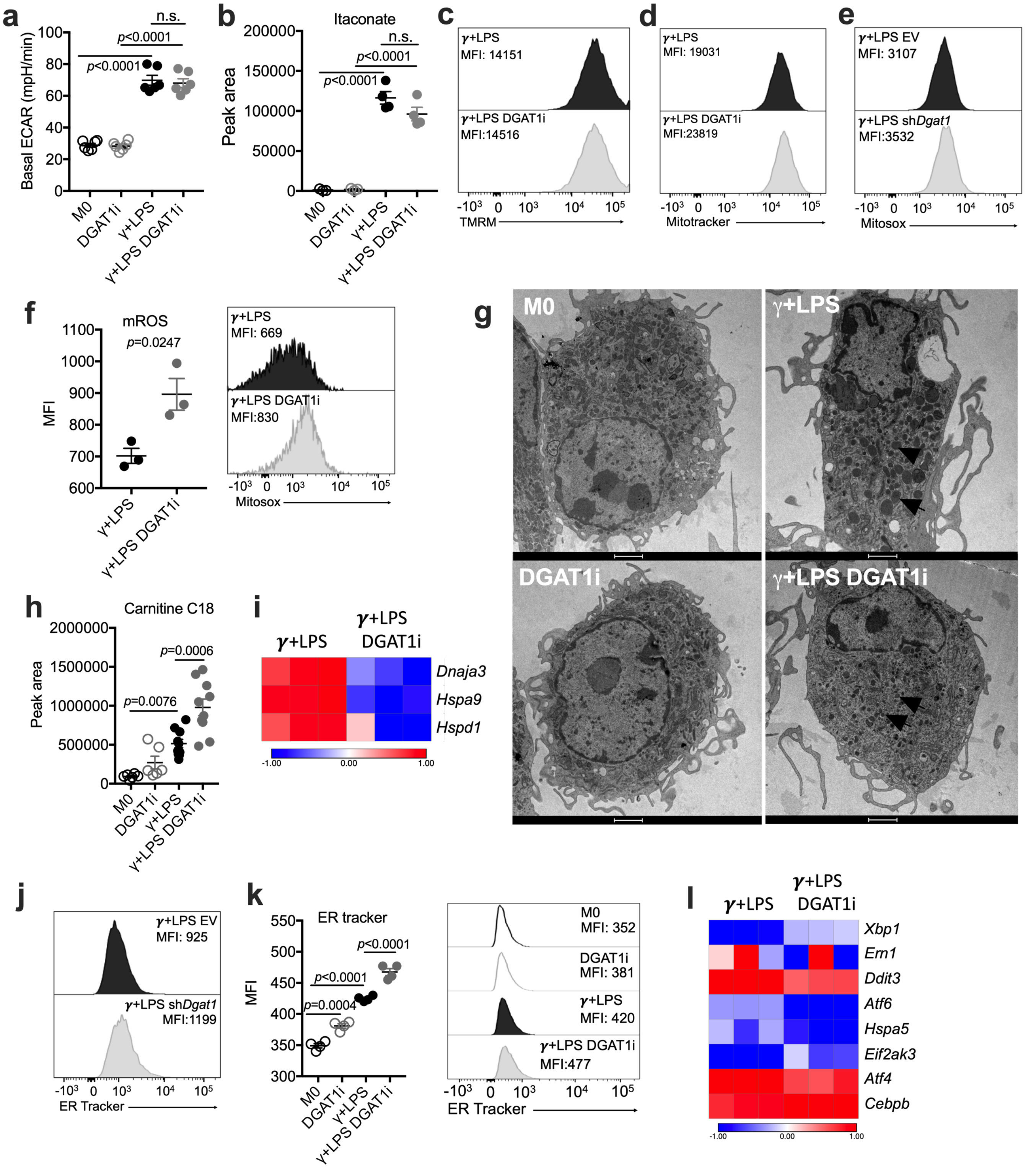

Supplementary Figure 3: Loss of function of DGAT1 does not change overall cell metabolism in inflammatory macrophages.

(a) Basal ECAR of macrophages cultured with IFNγ + LPS, or without (M0), with or without DGAT1i for 18 h (n = 6 biologically independent samples) and **(b)** total pool of itaconate (M0 and DGAT1i n = 3, γ + LPS and γ + LPS DGAT1i n = 4 biologically independent samples). **(c)**

Representative histogram of TMRM and **(d)** Mitotracker deep red staining in macrophages after 18 h of culture with IFNγ + LPS with or without DGAT1i, (MFI, median fluorescence intensity)**. (e)** Representative histogram of Mitosox staining in macrophages transduced with empty vector or sh*Dgat1* and cultured with or without IFNγ + LPS for 18 h**, (f)** Effects of DGAT1i on mitochondrial reactive oxygen species. Macrophages were cultured with IFNγ + LPS for 18 h (n = 3 biologically independent samples), and representative histogram. **(g)** Low magnification electron micrographs of resting (M0) macrophages or IFNγ + LPS-stimulated macrophages, cultured with or without DGAT1i for 18 h; arrows are pointing to lipid droplets (LD) (scale bars represent 2 μm). **(h)** Carnitine and C18-acylcarnitine in resting (M0) macrophages or IFNγ + LPS-stimulated macrophages, cultured with or without DGAT1i for 18 h (M0 and DGAT1i n = 6, γ + LPS and γ + LPS DGAT1 n = 10 biologically independent samples). **(i)** Endoplasmic reticulum (ER) stress response genes at 18 h post stimulation as measured using RNAseq. **(j)** Representative histogram of ER tracker staining in macrophages transduced with empty vector or sh*Dgat1* and cultured with or without IFNγ + LPS for 18 h. **(k)** ER mass in macrophages cultured with IFNγ + LPS, or without (M0), with or without DGAT1i for 18h (n = 4 biologically independent samples) and representative histogram. **(l)** Mitochondrial unfolded protein response genes at 18 h post stimulation with IFNγ + LPS without or with DGAT1i as measured using RNAseq. Data are represented as mean values ± s.e.m. One-way ANOVA with Bonferroni's multiple comparison test **(a-b, h, k)**; Unpaired two-tailed Student's t test **(f)**. (a-f, h, j, k) representative of tree experiments and (g, i, l) representative of one experiment. Source data are provided as a Source Data file.

Supplementary Figure 4

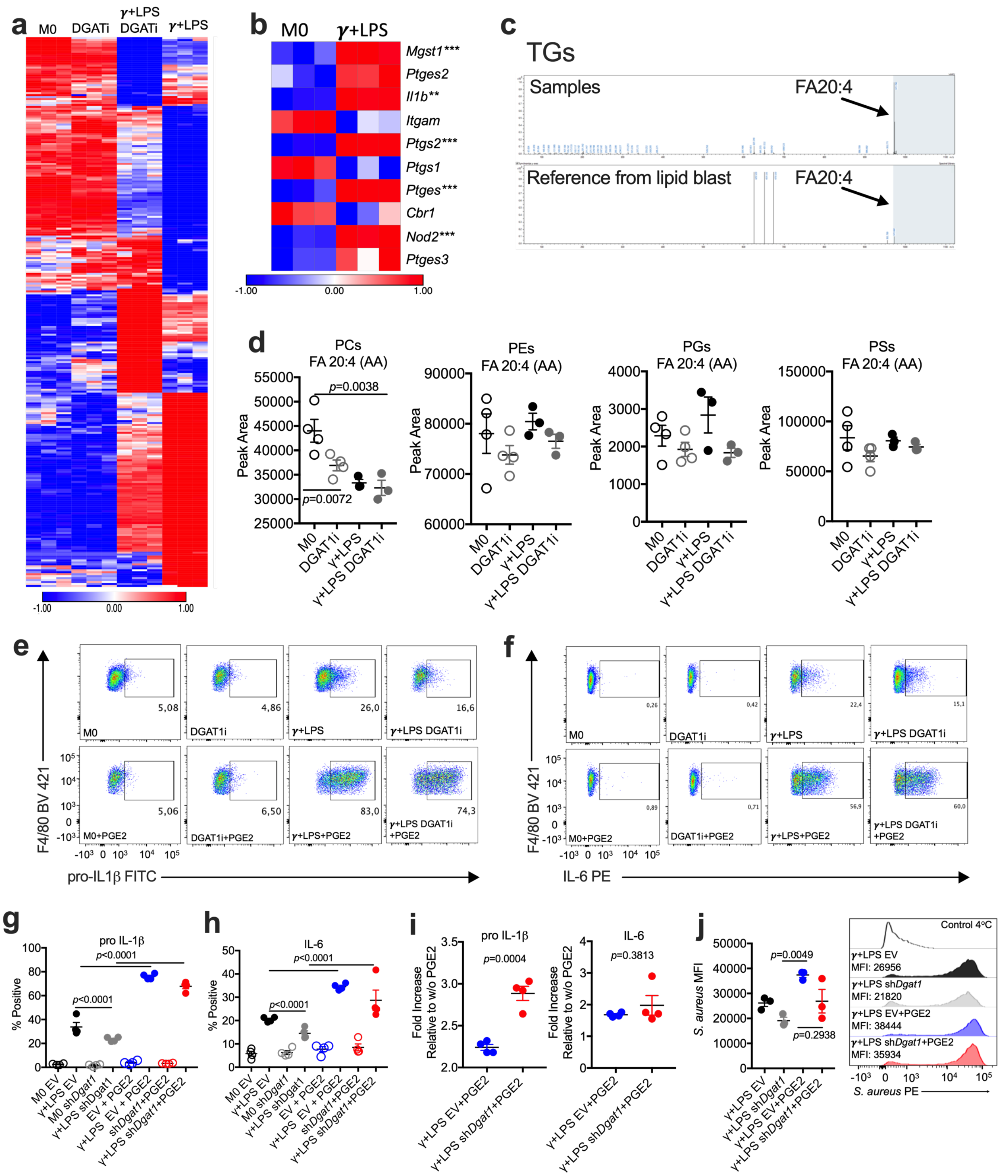

Supplementary Figure 4.: Down- and up-regulated genes and pathways in macrophages with loss of function of *DGAT1*.

(a) Significantly regulated (p<0.01) genes in macrophages stimulated without (M0) or with IFNγ + LPS for 18 h in the presence or absence of DGAT1i. 136 genes down-regulated and 149 genes up-regulated. Data from RNA-seq. **(b)** Prostaglandin-E synthase activity genes expression in resting (M0) and macrophages treated with IFNγ + LPS for 18 h (**p<0.01; ****p*<0.001) data from

RNA-seq. **(c)** Representative TGs mass spectrum (MS) of FA 20:4 (arachidonic acid (AA)) from IFNγ + LPS-stimulated macrophages cultured with or without DGAT1i for 18 h, x-axis :mass/charge ratio, y-axis: signal intensity. The MS shown is a combination of all the samples from this experiment. **(d)** PCs, PEs, PGs and PSs containing FA 20:4 (AA) at 18 h (M0 and DGAT1i n = 4, γ + LPS and γ + LPS DGAT1i n = 3 biologically independent samples). **(e)** pro-IL1β and **(f)** IL-6 representative intracellular staining in macrophages 6 h after stimulation with IFNγ + LPS with or without DGAT1i and/or exogenous PGE2. PGE2 was added 1 h after IFNγ + LPS or IFNγ + LPS + DGAT1i, and cells were collected 5 h later (values are percentages). **(g, h)** Macrophages were transduced with empty vector (EV) or sh*Dgat1* and cultured with or without IFNγ + LPS, with or without exogenous PGE2 (added 1 h after IFNγ + LPS) for 5 h. Pro IL-1β **(g)** or IL-6 **(h)** positive macrophages (percentage cytokine-positive F4/80$^+$ cells, are shown (n = 4 biologically independent samples). **(i)** Fold increases in pro IL-1β and IL-6 production (measured as percentage cytokine-positive F4/80$^+$ cells) induced by exogenous PGE2 in macrophages (n = 4 biologically independent samples). **(j)** Effect of PGE2 on the ability of EV- or sh*Dgat1*-transduced macrophages to phagocytose *S. aureus* (PE-labelled) after simulation with IFNγ + LPS. Data show MFI values of *S.aureus*-associated fluorescence in F4/80$^+$ cells (n = 3 biologically independent samples) and representative histograms, (MFI, median fluorescence intensity). Data are represented as mean values ± s.e.m. One-way ANOVA with Bonferroni's multiple comparison test **(d, g-h, j)**; Unpaired two-tailed Student's t test **(i)**. (a-d) representative of one experiment, (e-j) representative of two experiments. Source data are provided as a Source Data file.
