# Supplementary material for: Triacylglycerol synthesis enhances macrophage inflammatory function
Source: Nat Commun. 2020 Aug 14;11:4107. doi: 10.1038/s41467-020-17881-3 (PMC7427976; doi:10.1038/s41467-020-17881-3)
Supplement: Supplementary file 3 — Reporting Summary [file 41467_2020_17881_MOESM3_ESM.pdf]

nature research

Corresponding author(s): Edward J Pearce

Last updated by author(s): 2020/07/04

Reporting Summary

Nature Research wishes to improve the reproducibility of the work that we publish. This form provides structure for consistency and transparency in reporting. For further information on Nature Research policies, see Authors & Referees and the Editorial Policy Checklist .

Statistics

For all statistical analyses, confirm that the following items are present in the figure legend, table legend, main text, or Methods section.

n/a	Confirmed	
☐	☒	The exact sample size (n) for each experimental group/condition, given as a discrete number and unit of measurement
☐	☒	A statement on whether measurements were taken from distinct samples or whether the same sample was measured repeatedly
☐	☒	The statistical test(s) used AND whether they are one- or two-sided<br>*Only common tests should be described solely by name; describe more complex techniques in the Methods section.*
☒	☐	A description of all covariates tested
☐	☒	A description of any assumptions or corrections, such as tests of normality and adjustment for multiple comparisons
☐	☒	A full description of the statistical parameters including central tendency (e.g. means) or other basic estimates (e.g. regression coefficient) AND variation (e.g. standard deviation) or associated estimates of uncertainty (e.g. confidence intervals)
☐	☒	For null hypothesis testing, the test statistic (e.g. F, t, r) with confidence intervals, effect sizes, degrees of freedom and P value noted<br>*Give P values as exact values whenever suitable.*
☒	☐	For Bayesian analysis, information on the choice of priors and Markov chain Monte Carlo settings
☒	☐	For hierarchical and complex designs, identification of the appropriate level for tests and full reporting of outcomes
☒	☐	Estimates of effect sizes (e.g. Cohen's d, Pearson's r), indicating how they were calculated

Our web collection on statistics for biologists contains articles on many of the points above.

Software and code

Policy information about availability of computer code

Data collection	BD FACSDiva (version 8.0.1), Wave (version: 2.4), Agilent Mass Hunter(version B.08.00), Bruker TASQ Client (version:2.0), Bruker MetaboScape (version:4)
Data analysis	FlowJo (version: 10.1), Prism (version: 7), R (version: 3.4.2), Morpheus (online), ImageJ (version: 1.52), STAR (version: 2.5.2b-1), featureCounts (version: 1.6.0.1), DESeq2 (version 1.18.1)

For manuscripts utilizing custom algorithms or software that are central to the research but not yet described in published literature, software must be made available to editors/reviewers. We strongly encourage code deposition in a community repository (e.g. GitHub). See the Nature Research guidelines for submitting code & software for further information.

Data

Policy information about availability of data

All manuscripts must include a data availability statement. This statement should provide the following information, where applicable:

- Accession codes, unique identifiers, or web links for publicly available datasets
- A list of figures that have associated raw data
- A description of any restrictions on data availability

Next-generation sequencing data can be accessed at Gene Expression Omnibus under accession code GSE145523. The gene expression data set used in Fig. 1a and Supplementary Fig.1a and b is from19 deposited at NCBI GEO database and are publicly accessible under accession number GSE123596. The code used for palmitate tracing data processing is available from the authors. The source data underlying Fig 1b-g, 2a,c-r, 3a-g,i,j,l and 4c-h and Supplementary Figure1a-b,d,f-g; 2a,e-g,j,k, m, 3a-b,f,i,k and 4d,g-j are provided as a Source Data file.

Field-specific reporting

Please select the one below that is the best fit for your research. If you are not sure, read the appropriate sections before making your selection.

- [x] Life sciences
- [ ] Behavioural & social sciences
- [ ] Ecological, evolutionary & environmental sciences

For a reference copy of the document with all sections, see nature.com/documents/nr-reporting-summary-flat.pdf

Life sciences study design

All studies must disclose on these points even when the disclosure is negative.

Sample size	Predetermined sample sizes were not determined. Sample size were determined based on previous observations and publications in addition to feasibility of experiments.
Data exclusions	No data exclusion criteria were set, and no data were excluded from the analyses.
Replication	Experiments were repeated multiple times as described in figure legends and we used data only when differences between experimental groups were reproducibly different in multiple, independent experiments.
Randomization	Animals ordered from the breeding facility were assigned to cages by the facility staff without, who had to knowledge of experiments for which the mice were intended. We simply chose cages with already allocated mice, and these cages were randomly assigned to treatment groups.<br>In experiments performed on cells, mice were randomly assigned.
Blinding	Blinding was not performed as the majority of experiments were performed by a single individual (the first-author) making blinding not possible.<br>For in vivo IP LPS experiments blinding was unable to be performed as mice given IP LPS displayed visible symptoms, and T863 treatment visibly ameliorated these symptoms, making treatment groups obviously discernible.

Reporting for specific materials, systems and methods

We require information from authors about some types of materials, experimental systems and methods used in many studies. Here, indicate whether each material, system or method listed is relevant to your study. If you are not sure if a list item applies to your research, read the appropriate section before selecting a response.

Materials & experimental systems

n/a	Involved in the study	
[ ]	[x]	Antibodies
[ ]	[x]	Eukaryotic cell lines
[x]	[ ]	Palaeontology
[ ]	[x]	Animals and other organisms
[x]	[ ]	Human research participants
[x]	[ ]	Clinical data

Methods

n/a	Involved in the study	
[x]	[ ]	ChIP-seq
[ ]	[x]	Flow cytometry
[x]	[ ]	MRI-based neuroimaging

Antibodies

Antibodies used	The following antibodies for Flow cytometry were obtained from Biolegend, eBioscience and Thermofisher.<br>Extracellular staining:<br>CD11b (PEcy7, #101215-Biolegend, clone: M1/70, 1:300)<br>F4/80 (BV421 #123131-Biolegend, clone: BM8, 1:300)<br>F4/80 (APCcy7#123117-Biolegend, clone: BM8, 1:300)<br>Tim4 (APC, #129907-BioLegend, clone: F31-5G3, 1:200)<br>Live/Dead Fixable Blue (L23105-ThermoFisher, 1:500)<br><br>Intracellular staining:<br>pro-IL1b (FITC, #11-7114-82-eBioscience, clone: NJTEN3, 1:200)<br>IL-6 (PE, #504503-Biolegend, clone: MP5-20F3, 1:200)<br><br>Fluorescent dyes:<br>BODIPY™ 493/503 (#D3922-ThermoFisher, 2uM)<br>BODIPY® FL C16 (#D3821-ThermoFisher, 2uM)<br>MitoSOX™Red (#M36008-ThermoFisher, 5uM)

	Mitotracker™ Deep Red (#M22426-ThermoFisher, 50nM)<br>TMRM (#T668-ThermoFisher, 50nM)<br>ER Tracker™ green (#E34251-ThermoFisher, 1uM)<br>ER Tracker™ red (#E34250-ThermoFisher,1uM)
Validation	All antibodies and fluorescent dyes are commercially available and each company has validated each antibody for application and species use. Further information regarding validation approaches can be found at:<br>Biolegend: https://www.biolegend.com/en-us/reproducibility<br>eBiosciences and ThermoFisher: https://www.thermofisher.com/br/en/home/life-science/antibodies/invitrogen-antibody-validation.html

Eukaryotic cell lines

Policy information about cell lines

Cell line source(s)	HEK293Ts were sourced from ATCC
Authentication	Cell line was not authenticated
Mycoplasma contamination	Cell line tested negative for mycoplasma.
Commonly misidentified lines (See ICLAC register)	No commonly misidentified cell lines were used in the study.

Animals and other organisms

Policy information about studies involving animals; ARRIVE guidelines recommended for reporting animal research

Laboratory animals	C57Bl/6 mice (RRID: IMSR_JAX:000664) male, aged 6-8 weeks were used in this study. Mice were housed under controlled conditions, namely 20–21°C, 55–65% relative humidity, and 12:12 light-dark cycle. Food was available ad libitum for all animals.
Wild animals	Study did not involve wild animals
Field-collected samples	Study did not involve field-collected samples
Ethics oversight	All mice were maintained in specific pathogen-free conditions under protocols approved by the animal care committee of the Regierungspräsidium Freiburg, Germany, in compliance with all relevant ethical regulations.

Note that full information on the approval of the study protocol must also be provided in the manuscript.

Flow Cytometry

Plots

Confirm that:

- [x] The axis labels state the marker and fluorochrome used (e.g. CD4-FITC).
- [x] The axis scales are clearly visible. Include numbers along axes only for bottom left plot of group (a 'group' is an analysis of identical markers).
- [x] All plots are contour plots with outliers or pseudocolor plots.
- [x] A numerical value for number of cells or percentage (with statistics) is provided.

Methodology

Sample preparation	BM macrophages were polarized as described in the paper and harvested by gentle scraping. Peritoneal macrophages were harvested by peritoneal lavage with sterile PBS containing 2 mM EDTA. Single cell suspensions were kept at 4°C and blocked with 5 µg per mL anti-CD16/32 (Biolegend).
Instrument	BD LSRII and LSR Fortessa
Software	Data collection was performed using BD FacsDiva and analysis was performed with FlowJo
Cell population abundance	No sorting was performed, just flow cytometry
Gating strategy	All flow cytometry experiments used preliminary gates for: FSC singlets, SSC singlets, cell population of interest by FSC and SSC, then live cells based on viability dye exclusion. Following this, peritoneal macrophages were identified as Live, F4/80+, CD11b+, Tim4+. Further, F4/80+ TIM4neg and F4/80+ TIM4+ were gated for Bodipy 493/503. Bone marrow macrophages were identified as Live, F480+. Following this, macrophages were gated on Bodipy 493/503, Bodipy FL C16, ER Tracker, Mitosox, Mitotracker,

TMRM, L-6, pro-IL1b and S aureus.
Positive populations were determined relative to a biological control such as an unstained control (for Bodipy 493/503, Bodipy FL C16, ER Tracker, Mitosox, Mitotracker, TMRM), FMO for IL-6 and pro-IL1b and a control at 4oC for Phagocytosis assay.

☒ Tick this box to confirm that a figure exemplifying the gating strategy is provided in the Supplementary Information.
